# The semirecumbent position for high-resolution esophageal manometry. Results of a feasibility study

**DOI:** 10.1097/MEG.0000000000002143

**Published:** 2021-04-09

**Authors:** Stefano Siboni, Carlo G Riva, Davide Ferrari, Matteo Capuzzo, Emanuele Asti, Luigi Bonavina

**Affiliations:** Department of Biomedical Sciences for Health, Division of General and Foregut Surgery, IRCCS Policlinico San Donato, University of Milan, Milano, Italy

**Keywords:** body position, Chicago classification, esophageal motility disorders, gastroesophageal reflux disease, igh-resolution manometry, normative metrics, provocative testing

## Abstract

Normative values for high-resolution manometry (HRM) have been obtained with the patient lying supine. The aim of the study was to compare supine, semirecumbent and sitting positions during HRM in terms of variation in normative metrics, diagnostic yield, and patient’s comfort.

**Methods:**

A prospective, single-center feasibility study was planned in consecutive patients referred to the esophageal function laboratory. In each of the three positions, 10 consecutive 5 ml water swallows and three 10 ml multiple rapid swallows were administered. Validated reflux questionnaires were administered prior to the test, and a visual analogue scale (VAS) assessing the patient’s comfort after the test.

**Results:**

Twenty patients presenting with gastroesophageal reflux symptoms completed the study protocol. The intra-abdominal segment of the lower esophageal sphincter was significantly longer in the sitting position (*P* = 0.013), and the multiple rapid swallow distal contractile integral was lowest in the supine position (*P* = 0.012). The VAS comfort score did not significantly differ in the three body positions (*P* = 0.295). The concordance in the final diagnosis was 80% for semirecumbent vs. sitting (kappa = 0.15; *P* = 0.001), 70% for supine vs. sitting and 65.0% for semirecumbent vs. supine.

**Conclusion:**

Compared to the supine position, both the semirecumbent and sitting position seems to provide similar advantages. HRM metrics and the final manometric diagnosis may be affected by body position, but complementary maneuvers, such are the rapid drink challenge, can resolve diagnostic discrepancies and improve the overall accuracy of the test.

## Introduction

Esophageal motility disorders are currently classified using high-resolution manometry (HRM) and the categories provided by the Chicago Classification (CC v3.0). According to CC v3.0, ten 5-ml water swallows are analyzed in the supine position in patients with no prior esophago-gastric junction surgery [1]. However, real-world clinical practice is not totally respectful of these guidelines [2]. More recently, provocative tests, including multiple rapid swallows, rapid drink challenge, solid meal swallows and straight leg raise maneuver have been added to improve the accuracy of HRM [3–6]. Variations in methodology among institutions may affect the intrinsically complex relationship between symptoms and HRM metrics and, indirectly, the reliability of the test [7]. Last but not least, the overall reproducibility of HRM has been questioned based on the finding of significant changes of diagnosis over repeat HRM studies [8].

Surprisingly, now that the HRM catheter does not need to be at the level of pressure transducers as it was the case with perfusion manometry [9], the test is still performed with the patient lying in the supine position. Normative values have been obtained with HRM in the upright but not in the semirecumbent position. The aim of this pilot study was to compare HRM examinations consecutively performed in the supine, semirecumbent and sitting position in a group of symptomatic patients referred to the esophageal function laboratory.

## Methods

A prospective, single-center feasibility study was performed to compare the effects of supine, semirecumbent and sitting position on HRM metrics, final manometric diagnosis and patient’s comfort. The study protocol conforms to the ethical guidelines of the 1975 Declaration of Helsinki (6th revision) as reflected in a priori approval by the institutional review board. Informed consent was obtained from each patient included in the study

### Study protocol

**Fig. 1.**
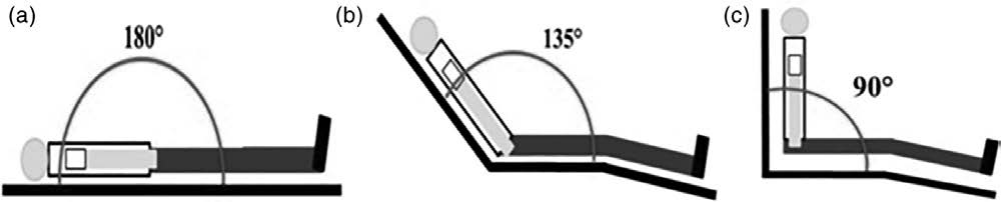
Body positions according to the high-resolution manometry protocol: (a) supine, (b) semirecumbent and (c) supine.

The HRM study was performed by sequentially placing the patient in the supine position, in the semirecumbent position (135° angle between the seat and the seatback), and in the sitting position (Fig. 1). Consecutive patients undergoing esophageal HRM in our esophageal function laboratory were asked to participate in the study. Study outcomes were the observed changes of different body positions on HRM metrics, the concordance between positions in terms of final manometric diagnosis according to the CC v3.0 criteria, and the degree of patients’ comfort in each body position. Demographic data, relevant medical history, previous esophago-gastric surgery, present foregut symptoms, duration of symptoms and previous/current treatment with proton pump inhibitors (PPI) were recorded. Symptoms were assessed using the Gastro-Esophageal Reflux Disease (GERD) health-related quality of life (HRQL) [10], the GERD-Q [11] and the Reflux Symptom Index [12] questionnaires. A 10-point visual analogue scale was used to assess the degree of patient’s comfort (1 = no discomfort; 10 = maximum discomfort) on each body position assumed during the test.

### High-resolution manometry

The HRM was performed using a solid-state catheter with 36 circumferentially incorporated sensors spaced at 1-cm intervals (Medtronic, Duluth, Georgia, USA). All patients were instructed to fast at least 6 h before the exam and to avoid drugs with known potential effects on esophago-gastric motility. The catheter was inserted trans-nasally, and a 5-min period of adaptation was allowed before starting the test. Baseline measurements were taken during 30 s while the patient refrained from swallowing. In the supine position, 10 consecutive swallows of 5 ml of natural water at room temperature were administered by a syringe every 30 s. Three multiple rapid swallows (MRS) were performed with five consecutive boluses of 2 ml water administered at <4 s intervals. Then, the patient was switched to the semirecumbent position without moving the catheter. After a 5-min period of adaptation, the baseline was recorded and the same procedure was repeated. Finally, the patient was moved to the sitting position, and the same procedure was repeated again. The measured HRM variables for each position were the following: lower esophageal sphincter (LES) basal characteristics, including total and intra-abdominal length, pressure, distance in cm between LES and crura diaphragm, integrated relaxation pressure, distal esophageal amplitude, distal latency, distal contractile integral (DCI) and intrabolus pressure. The contractile reserve was assessed by MRS, and a ratio of MRS DCI to mean single swallow DCI >1 indicated the presence of adequate physiological reserve. Moreover, the percentage of failed, weak and premature swallows in each position was recorded.

### Data analysis

Four physicians (S.S., C.G.R., D.F. and M.C.) performed the procedures. The HRM metrics were analyzed using ManoView (Covidien/Given Imaging, Duluth, Georgia, USA) with updated software for CC v3.0 [1]. The references for the normal range were the CC v3.0 criteria and the Lyon consensus [13]. A manometric diagnosis was made in each position, and the results were compared. In case of uncertain diagnosis, the senior author (L.B.) was contacted to solve the doubt. The rate of agreement of the final diagnosis among the different body positions was calculated.

### Statistical analysis

Categorical data are reported using frequencies and proportions. Continuous data are reported as mean ± SD or median and interquartile range, as appropriate. Variables between the three groups were compared using one-way repeated measure ANOVA test. Kappa Cohen’s test was used to evaluate the agreement between the diagnoses in the different body positions (semirecumbent vs. supine, semirecumbent vs. sitting and supine vs. sitting position).

Differences were considered statistically significant when *P* value <0.05. Statistical analysis was performed using the Statistical Package for Social Sciences software version 23.

## Results

Between October 2019 and October 2020, a total of 20 patients were included in the study. All patients underwent HRM for typical and atypical reflux symptoms. Ten patients had previous endoscopic and/or radiologic diagnosis of hiatal hernia, and five had esophagitis at the last endoscopy. None of the patients complained of dysphagia. The majority of patients were on PPI therapy. The complete demographic and clinical data are reported in Table 1.

**Table 1.** Demographics and clinical presentation

	Patients (*n* = 20)
Age, y, mean ± SD	54 ± 13.8
Male, *n* (%)	10 (50.0)
BMI (kg/m^2^), mean ± SD	24.1 ± 2.7
Endoscopic findings	
Esophagitis, *n* (%)	5 (25.0)
Barrett’s esophagus, *n* (%)	2 (10.0)
Hiatal hernia, *n* (%)	10 (50.0)
Symptoms	
Heartburn, *n* (%)	10 (50.0)
Regurgitation, *n* (%)	9 (45.0)
Epigastric pain, *n* (%)	3 (15.0)
Cough, *n* (%)	3 (15.0)
Sore throat, *n* (%)	3 (15.0)
Dysphagia, *n* (%)	0
Use of PPI, *n* (%)	16 (80.0)
PPI full responders, *n* (%)	6 (30.0)
Previous antireflux surgery	
MSA, *n* (%)	3 (15.0)
Nissen fundoplication, *n* (%)	1 (5.0)
Questionnaires	
GERD-HRQL, mean ± SD	9.8 ± 7.8
GERDQ-A, mean ± SD	4.9 ± 4.5
GERDQ-B, mean ± SD	1.5 ± 1.9
RSI, mean ± SD	9.5 ± 9.6

GERD-HRQL, Gastro-Esophageal Reflux Disease - Health Related Quality of Life; GERD-Q, GERD Questionnaire; MSA, magnetic sphincter augmentation; PPI, proton-pump inhibitors; RSI, reflux symptom index.

All studies were successfully completed in the three body positions. Table 2 shows the comparison of the HRM variables in the supine, semirecumbent and sitting positions, respectively. There was a significant increase in the LES intrabdominal length with the patient in the sitting position (*P* = 0.004). The mean DCI after MRS progressively increased from the supine to the sitting position (*P* = 0.012). Overall, more patients were diagnosed with ineffective esophageal motility (IEM) in the semirecumbent and in the sitting position, whereas more patients with absent peristalsis were diagnosed in the supine position (Table 3). The mean ± SD comfort score did not significantly differ among groups (4.0 ± 2.1 supine vs. 2.0 ± 1.2 semirecumbent vs. 2.5 ± 1.0 sitting, *P* = 0.295).

**Table 2.** Comparison of high-resolution manometry variables recorded in three different body positions

Variable	Supine	Semirecumbent	Sitting	*P* value
LES length (cm), mean ± SD	1.51 ± 0.26	1.58 ± 0.23	1.61 ± 0.19	0.126
LES intrabdominal length (cm), mean ± SD	0.33 ± 0.47	0.24 ± 0.33	0.83 ± 0.69	0.004
Hiatal hernia, *n* (%)	10 (52.6)	8 (42.1)	5 (26.3)	0.114
Hiatal hernia size (cm), mean ± SD	2.2 ± 1.5	2.1 ± 1.2	2.1 ± 1.6	0.125
LES basal pressure (mmHg), mean ± SD	15.3 ± 11.8	17.5 ± 9.6	14.5 ± 11.5	0.065
IRP (mmHg), mean ± SD	6.3 ± 6.1	6.5 ± 5.6	4.9 ± 4.7	0.134
LES % of relaxation, mean ± SD	60.3 ± 21.7	57.8 ± 38.4	72.2 ± 39.2	0.136
DCI (mmHg-cm-s), mean ± SD	1655.7 ± 1141.1	1161.9 ± 868.2	1304.4 ± 1184.4	0.052
MRS DCI (mmHg-cm-s), mean ± SD	921 ± 865.7	1273.5 ± 1049	1763.9 ± 1643.3	0.012
MRS ratio, mean ± SD	0.9 ± 0.6	1.4 ± 0.6	2 ± 1.6	0.066
IBP (mmHg), mean ± SD	11.3 ± 4.6	12.2 ± 4.5	12.3 ± 3.9	0.332
Distal latency (s), mean ± SD	5.9 ± 0.9	6.3 ± 1.1	6 ± 0.8	0.159
% failed swallows, mean ± SD	19.5 ± 36.6	19.5 ± 35.8	19.5 ± 29.9	0.742
% weak swallows, mean ± SD	7.4 ± 15.2	16.8 ± 22.9	15.8 ± 20.6	0.164
% successful swallows, mean ± SD	63.2 ± 40.3	63.2 ± 37.7	54.2 ± 40.2	0.620
% premature contraction, mean ± SD	6.3 ± 20.9	1.1 ± 3.2	1.1 ± 4.6	0.149

DCI, distal contractile integral; IBP, intrabolus pressure; IRP, integrated relaxation pressure; LES, lower esophageal sphincter; MRS, multiple repeated swallows.

**Table 3.** Final high-resolution manometry diagnosis according to the different body positions

Variable	Supine	Semirecumbent	Sitting
Normal, *n* (%)	13	14	13
IEM, *n* (%)	1	4	5
Absent peristalsis, *n* (%)	3	2	1
DES, *n* (%)	1	0	1
Jackhammer esophagus, *n* (%)	1	0	0
EGJ-OO, *n* (%)	1	0	0

IEM, ineffective esophageal motility; DES, distal esophageal spasm; EGJ-OO, esophago-gastric junction outflow obstruction.

**Fig. 2.**
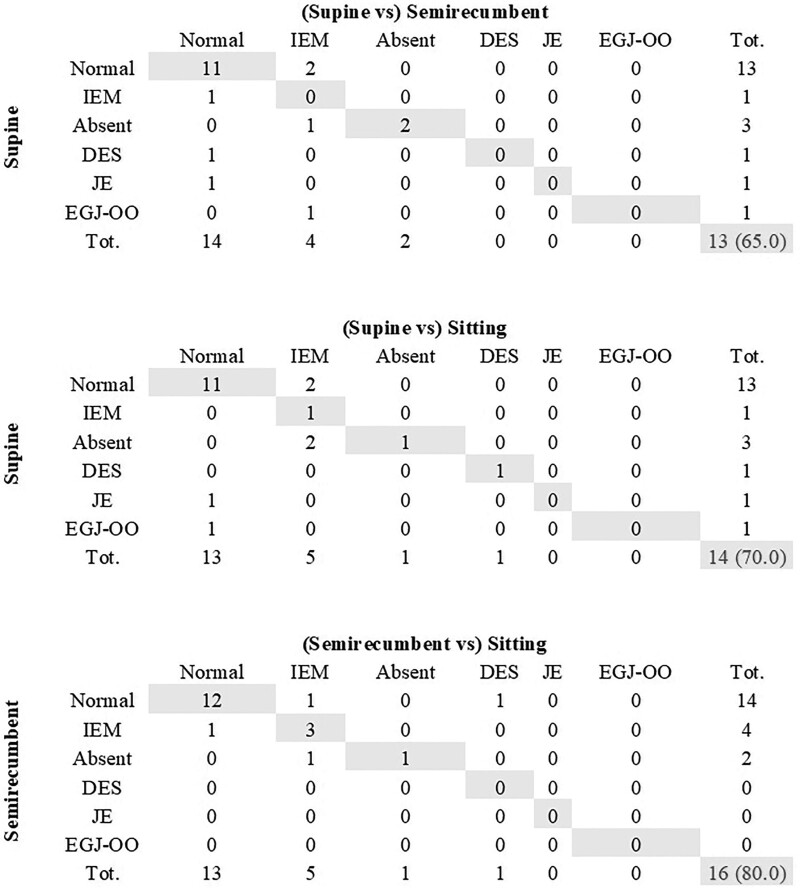
Concordance of manometric findings among different body positions. Grey boxes represent number of cases with the same diagnosis in two different positions. DES, distal esophageal spasm; EGJ-OO, esophago-gastric junction outflow obstruction; IEM, ineffective esophageal motility; JE, Jackhammer esophagus

Figure 2 shows the concordance of the final manometric diagnosis for the different body positions: the highest value was found between semirecumbent and sitting position (80.0%, kappa = 0.15, fair value of concordance, *P* = 0.001), whereas the concordance between supine and semirecumbent and between supine and sitting position was 65.0% (kappa = 0) and 70.0% (kappa = 0), respectively.

## Discussion

The findings of the current study indicate that both the semirecumbent and the sitting position represent a valid alternative to the supine position in performing esophageal HRM. Even if HRM metrics vary according to the different positions and may affect the final manometric diagnosis, the concordance between the semirecumbent and the sitting position was 80%. Although there was no significant difference in the degree of patient’s comfort during HRM, both the semirecumbent and the sitting position simulate more closely a real-life behavior and allow to safely perform some provocative maneuvers. It has previously been suggested that HRM should preferably be performed in the sitting position, especially in patients with swallowing difficulties and risk of aspiration, and that adjunctive maneuvers, such as the rapid drink challenge, should be added to resolve diagnostic discrepancies and improve overall accuracy [14]. Moreover, two previous studies, one in GERD patients [15] and one in normal volunteers [16] have reported the use of the semirecumbent position during HRM.

A recent systematic review including 1692 patients studied both in the supine and in the sitting position concluded that the sitting position can affect HRM metrics and may change the final manometric diagnosis [17]. In the present study, no significant differences in HRM metrics were found between supine, semirecumbent and sitting position, except that the mean LES intra-abdominal length was longer in the sitting position and the mean MRS-DCI progressively increased from the supine to the semirecumbent and then to the sitting position. We also found that the concordance of the final manometric diagnoses was greatest in the semirecumbent vs. the sitting position. In our study, IEM was more common in the semirecumbent and sitting position, possibly due to the lower DCI values registered in these positions. Ineffective esophageal motility has some relevance for surgeons who tailor the antireflux procedure according to the strength of esophageal motility [13]. Although changing body position can affect the motility pattern and may translate into a disagreement of the final manometric diagnosis, the clinical significance of this observation remains to be clarified. A recent study by Triadafilopoulos *et al*. [8] showed that a proportion of IEM patients did not retained the same diagnosis at a repeated HRM 15 months later. Our results did not differ from those of Misselwitz *et al*. [18] who reported that the concordance between supine and sitting positions increased from 67 to 90% when only the major motility disorders were considered.

Of note, the present study was performed according to the CCv3.0. The new, just published Chicago Classification has modified the HRM protocol by including supine and upright positions and provocative maneuvers such as the MRS and the rapid drink challenge test [19]. In fact, the standard protocol of 10 supine wet swallows may be insufficient and may overestimate the diagnosis of esophagogastric junction outlet obstruction and hypercontractile esophagus. Furthermore, especially in patients with a clear diagnosis of achalasia and at risk of aspiration, the upright position is preferable [20]. Interestingly, a Delphi European consensus study found no consensus that supine is the preferable position for HRM, although normative values and diagnostic guidelines were generated for that position [21].

### Study limitations

This was a feasibility study on a small sample size of symptomatic patients. It would have been interesting to include patients with dysphagia and achalasia. A rapid drink challenge test was not performed in any of the patients

The results of the present study seem to confirm that both the semirecumbent and sitting position provides similar advantages compared to the supine position during HRM. Posture is indeed a factor influencing HRM metrics and the final manometric diagnosis. However, appropriate reference values for the semirecumbent position need to be established. It is possible that a personalized HRM protocol, using a more comfortable body position and combining provocative tests, may increase the diagnostic yield of the test and the patient’s compliance.

## Acknowledgements

Work supported by AIRES (Associazione Italiana Ricerca ESofago).

## Conflicts of interest

There are no conflicts of interest.
